# Multi-stability of circadian phase wave within early postnatal suprachiasmatic nucleus

**DOI:** 10.1038/srep21463

**Published:** 2016-02-19

**Authors:** Byeongha Jeong, Jin Hee Hong, Hyun Kim, Han Kyoung Choe, Kyungjin Kim, Kyoung J. Lee

**Affiliations:** 1 Department of Physics, Korea University, Seoul, 136-713, Korea; 2 School of Biological Sciences, Seoul National University, Seoul 151-742, Korea

## Abstract

The suprachiasmatic nucleus (SCN) is a group of cells that functions as a biological master clock. In different SCN cells, oscillations of biochemical markers such as the expression-level of clock genes, are not synchronized but instead form slow circadian phase waves propagating over the whole cell population spatio-temporal struc- ture is a fixed property set by the anatomy of a given SCN. Here, we show that this is not the case in early postnatal SCN. Earlier studies presumed that their Based on bioluminescence imaging experiments with Per2-Luciferase mice SCN cultures which guided computer simulations of a realistic model of the SCN, we demonstrate that the wave is not unique but can be in various modes including phase- coherent oscillation, crescent-shaped wave, and most notably, a rotating pinwheel wave that conceptually resembles a wall clock with a rotating hand. Furthermore, mode transitions can be induced by a pulse of 38.5 °C temperature perturbation. Importantly, the waves support a significantly different period, suggesting that neither a spatially-fixed phase ordering nor a specialized pacemaker having a fixed period exist in these studied SCNs. These results lead to new important questions of what the observed multi-stability means for the proper function of an SCN and its arrhythmia.

The SCN of the hypothalamus^1^ times all of the daily rhythms such as the sleep-wake cycle, body temperature oscillations, and hormone release cycles. For mammals, it is composed of thousands of clock cells, specialized neuronal cells, that are capable of maintaining a circadian rhythm independently from one another: The firing rate of spontaneous neural spikes as well as the levels of various clock gene expressions and cytosolic calcium concentration oscillate in a circadian fashion^2^,^3^,^4^,^5^,^6^,^7^. They are not homogeneous, each having a quite different endogenous period that approximately ranges from 20 to 28 h^3^,^5^. In addition, within an SCN subregions can be identified based on their peptidergic contents^8^,^9^,^10^. Nevertheless, recent studies have demonstrated that within the SCN various cell-to-cell connectivities exist^11^,^12^,^13^,^14^,^15^ and they bring a coherence to the SCN, enabling it to be an organized master clock. Here, the term “coherence” involves two different issues^16^. On one hand, it represents the degree of frequency-locking (i.e., period-coherence), in which the different endogenous periods of clock cells are detuned to become, more or less, one single period. On the other hand, it refers to the degree of phase dispersal in space (i.e., phase-coherence). As for the phase-coherence, recent studies have shown that some circadian phase waves exist within the SCN^13^,^17^,^18^,^19^,^20^,^21^,^22^. However, their physical properties varied widely by case. Some important questions follow immediately. Do SCN phase waves have a (or have no) preferred shape, wavelength, or similarly, a preferred degree of phase-dispersal over the whole SCN? Do the properties of SCN waves correlate with the anatomy of the SCN? Importantly, is there a wave organizing center like the cardiac pacemakers in the heart? So far, the answers to these questions are largely unknown, but all previous studies tend to assume that the spatio-temporal structure of the wave is set by the anatomy of a given SCN. For example, Mieda *et al.*^23^ suggests that the dorsal area of the SCN functions as a phase-leading center and Lee *et al.*^24^ suggests that a rather extensive network of (neuromedin S-producing) clock cells within an SCN serves as a pacemaker.

In this article, using long-term, real-time luciferase reporter gene imaging and carefully controlled temperature perturbations on organotypic cultures of SCN slices, we carefully answer the raised questions. We demonstrate that even for a given SCN, the circadian wave can take several different forms that are stable when left unperturbed: They include phase-synchronized oscillation, planar or oval-shaped traveling waves, and most surprisingly, a steadily rotating pinwheel. The rotating pinwheel can be induced by a carefully timed perturbation, exploiting the non-monotonic phase (shifting) response nature of SCN clock cells. A specific mode is chosen by the history of the system and the different types of waves maintain a significantly different circadian periods. Taken all together, we have demonstrated that there is neither a topographically-fixed circadian phase ordering nor a phase-leading center within the SCN. Our key experimental findings are successfully recapitulated with numerical simulations with a mathematical model of SCN.

## Results

### Phase response curve (PRC) based on homogeneous temperature perturbations

**Figure 1:**
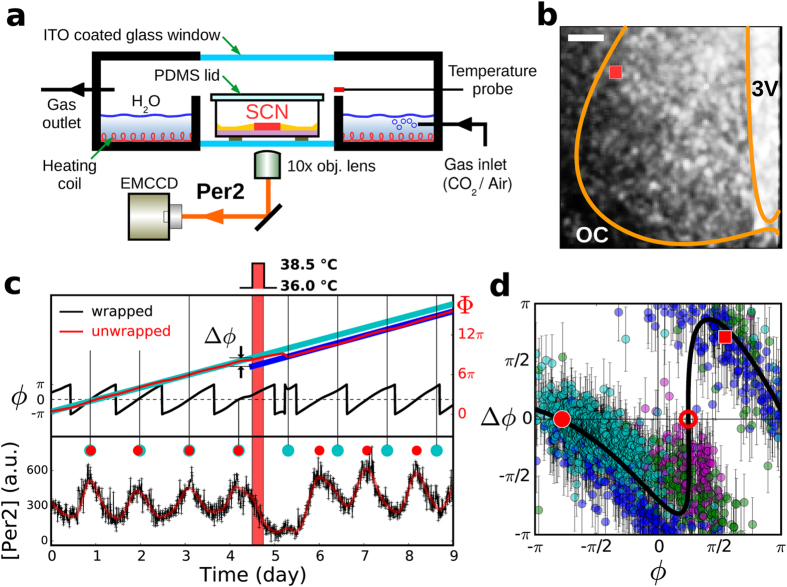
A representative phase response curve of SCN clock cell population based on temperature perturbations. (**a**) A home-built incubator for live SCN bioluminescence imaging. (**b**) A temporally averaged [Per2] luminescence image (scale bar: 100 *μm*; OC: optic chiasm; 3V: third ventricle). (**c**) A representative local [Per2] time series (in bottom frame, black: raw data; red: time series smoothed locally with a third order polynomial fit) and its 2*π*-wrapped (black line, top frame) and 2*π*-unwrapped phase (red line, top frame). The red vertical bar represents the duration of perturbation, and the red dots mark the positions of local [Per2] maxima (or *ϕ* = 0) while the cyan dots are projected maxima positions assuming the perturbation had not been given. The cyan line and blue line are a linear fit to the unwrapped phase before and after the perturbation, respectively. (**d**) The PRC of a cultured SCN based on four rounds of temperature perturbations. The four coloured (sequentially in time, violet, green, blue, cyan) clusters of dots match the four perturbations delivered in succession. The error bars in (**d**) are the uncertainty originating from the errors associated with the linear least-square fits of Φ, one before and the other after the perturbation: For the sake of clarity, only the grid points having an error bar less than 0.3*π* are included in the plot. Also, those grid points, whose period change more than 5 h with the perturbation, were discarded. The red square in (**b**) as well as (**d**) corresponds to the particular example shown in (**c**). The black solid line in (**d**) is a spline fit to the data points for a visual guide.

The experiments were conducted with brain slice (coronal section) cultures of PER2::LUC mutant mice containing an SCN. The culture slices were maintained continuously, often over a month, within a lab-built incubator, while their bioluminescence indicating the level of Per2 expression was being followed by an electron-multiplying charge-coupled device (EMCCD), as is schematically illustrated in Fig. 1a. A representative bioluminescence time series taken at a grid point (red square symbol in Fig. 1b) is shown with black crosses in the bottom frame of Fig. 1c, and the overlaid solid red line represents locally-smoothed data. Then, a Hilbert transformation^25^ was applied to the smoothed data to obtain its matching phase value [*ϕ* (2*π*-wrapped, black line) or Φ (2*π*-unwrapped, red line)], as in the top frame of Fig. 1c. The phase *ϕ* as well as the amplitude variation clearly supports a circadian oscillation in the cytosolic Per2 level.

For the purpose of disturbing the SCN circadian rhythm, we employed a temperature perturbation^26^,^27^,^28^ since it could be controlled precisely and reversibly at ease–and most important of all, it was found to be very effective. The system temperature was increased to 38.5 °C from 36.0 °C for 6 h as a perturbation, and it was sufficient enough for shifting the circadian phase, as shown in Fig. 1c (bottom) on which the red vertical bar represents the perturbation and red dots mark the actual positions of local amplitude maxima, whereas the cyan dots mark the projected positions of maxima based on the oscillation before the perturbation was delivered. For a systematic estimation of the phase shift Δ*ϕ* induced by the perturbation, we calculated the phase difference between the linear fit of Φ after the perturbation was delivered (blue line) and that of the projected Φ (cyan line), at the onset of the perturbation. Approximately 4 circadian cycles of data were used for each fit; 6 h before and after the perturbation were excluded from the analysis.

We found Δ*ϕ* to be a highly non-monotonic (flipped N-shaped) function of *ϕ* as shown in Fig. 1d. In general, at a given instance of time there existed a significant degree of circadian phase dispersal across an SCN. Consequently, a range of different Δ*ϕ* would be elicited in the phase-dispersed clock cell population after a temperature shock was given. Four temperature shocks were delivered when the system was in a different *ϕ* regime. And Δ*ϕ* was estimated for each grid point after each shock was delivered and plotted all together to reveal the *local* phase response curve (PRC) that is shown in Fig. 1d. The degree of phase-dispersal in space changed with each perturbation and the four temperature shocks were more than sufficient to cover the full 2*π* range of PRC. Surprisingly, the dynamic range of Δ*ϕ* was also a full cycle of 2*π*. The exemplary case analyzed in Fig. 1c constitutes a single point (red square symbol, Δ*ϕ* = 0.64*π*) in Fig. 1d.

The non-monotonic PRC carries a pair of fixed points, one of which is stable (filled red circle) and the other unstable (open red circle) [see Supplementary Fig. S1 for their functional roles], and it was found to be a common feature of all SCNs that we have examined (*n* = 6), although there existed some significant variations with regard to the dynamic range of Δ*ϕ* and the positions of the fixed points, from one SCN (animal) to the others. As we will discuss next, the highly non-monotonic nature of the PRC can confer some striking changes in the phase dispersal within an SCN.

### Formation of a rotating pinwheel wave

**Figure 2:**
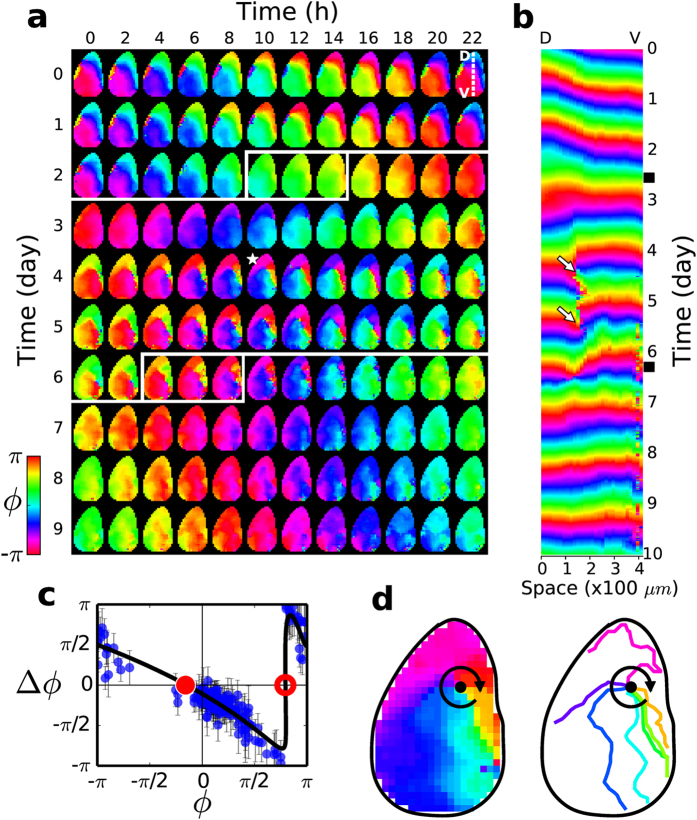
Formation and resetting of a pinwheel wave in a cultured SCN. (**a**) A sequence of phase images taken at every 2 h. The two, white-line bounded, rectangular boxes represent a perturbation at specific time and duration. (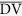 = 418*μm*) (**b**) One dimensional space (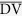) vs. time plot visualizes the formation of a phase singularity (marked by white arrows) following a perturbation, and its termination following another perturbation. Two black boxes on the time axis mark the two perturbations in sequence. (**c**) The PRC of the SCN under study, obtained based on the first perturbation leading to the pinwheel state (The black solid line is a spline fit of the data for visual guide). (**d**) A blown-up phase map [day 4, h 10, marked by a star in (**a**)] showing a phase singularity (black dot) around which a distorted pinwheel wave is formed. To its right are equi-phase lines taken every *π*/4. The horizontal position of 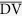 was chosen to cut through the position of the phase singularity. The position of the phase singularity was identified according to the algorithm given in ref. 43.

It was typical of our cultured SCN to support phase waves and one example is given in Fig. 2a (see day 0 and day 1): A crescent-shaped phase wave starts out from the dorsal (D) region to propagate toward the ventral (V) region, and it repeats stably in a circadian fashion (see Supplementary Movie 1). The period of this phase wave is measured to be 23.5 ± 0.6 h (mean ± SD). We note that the phase velocity is not uniform in space, being significantly higher in the ventrolateral region (core) than in the dorsomedial region (shell) immediately neighboring the 3V region (see Supplementary Fig. S2a). The one-dimensional space-time plot shown in Fig. 2b also well illustrates the wave properties. Subsequently, we delivered one of the aforementioned temperature perturbations during day 2, as marked by a white box in Fig. 2a. The particular time window (10 to 16 h) was chosen so that the range of the circadian phase *ϕ* covered by the SCN would include the unstable fixed point of its PRC. Thereby, the initial phase dispersal would be expanded greatly after the perturbation was delivered and possibly a new wave state could be established.

Indeed, just one properly timed perturbation was enough to produce the full 2*π* spectrum of Δ*ϕ* and to cover much of its “flipped N-shaped” PRC, including two fixed points as shown in Fig. 2c. Strikingly, the perturbation resulted in a rotating pinwheel in space, as is shown by the sequence of snapshot images in Fig. 2a (day 3 ~ 5) (also, see Supplementary Movie 1). As clearly illustrated in Fig. 2d, the pinwheel state is organized around a phase singularity (marked by a black dot), around which the phase completes a full 2*π* turn. Its existence is also evident in the 1D space-time plot of Fig. 2b (as marked by arrows). As the pinwheel rotates in the clockwise direction, the phase velocity changes, again much higher in the core region than in the shell region. It is particularly slow in the far right-hand side [see Supplementary Fig. S2b]. Interestingly, the period of the pinwheel 25.6 ± 0.1 h (mean ± SD) is significantly longer than that of the previous wave. The pinwheel state seemed quite stable if left undisturbed. Yet, by applying the same temperature perturbation (as marked by a white box during the day 6) we could easily remove the singularity and make the system oscillate in an almost perfect synchrony [see Fig. 2a (day 8 ~ 9) and its matching 1D space-time plot of Fig. 2b]. The newly established oscillatory state was very robust even after another perturbation was given with a period of 25.8 ± 0.9 h (mean ± SD).

A properly-timed temperature perturbation could produce some phase singularities. But, creating and keeping them stably for a long period of time were difficult. In fact, we achieved the pinwheel wave state only twice out of eight attempts with the same temperature perturbation, based on six different slices from six different animals. For its creation, first of all, one needs to obtain the complete PRC of the given sample, wait for the phases of clock cells to reach around the unstable fixed point of the PRC (as in the left frame of Fig. S1a), then give a perturbation. The perturbation that we have employed, however, does not always guarantee a birth of phase singularity. Second of all, it may be necessary for the system to have some small localized inhomogeneities about which the initial wave front can be broken, as often is the case for the formation of spiral waves in excitable media^29^. In a sense, the same perturbation can enhance the strength of localized phase inhomogeneities which are otherwise entrained by the bulk phase wave supported by the system. Incidently, the existence of small localized phase inhomogeneities in SCN has often been discussed in several earlier reports^13^,^17^,^30^. Alternatively, we can attribute the formation of phase singularities to a “front instability” of moving wave front^31^. Nevertheless, the exact underlying mechanism is unclear at the moment. We also observed that after their births phase singularities could drift significantly in space and move out of the system boundary. Overall, pinwheel states seemed to be less favored than other types of waves having no phase singularity associated. In fact, without a perturbation we never had a case of the pinwheel wave state naturally occurring.

### Formation and disappearance of localized domains oscillating out-of-phase

**Figure 3:**
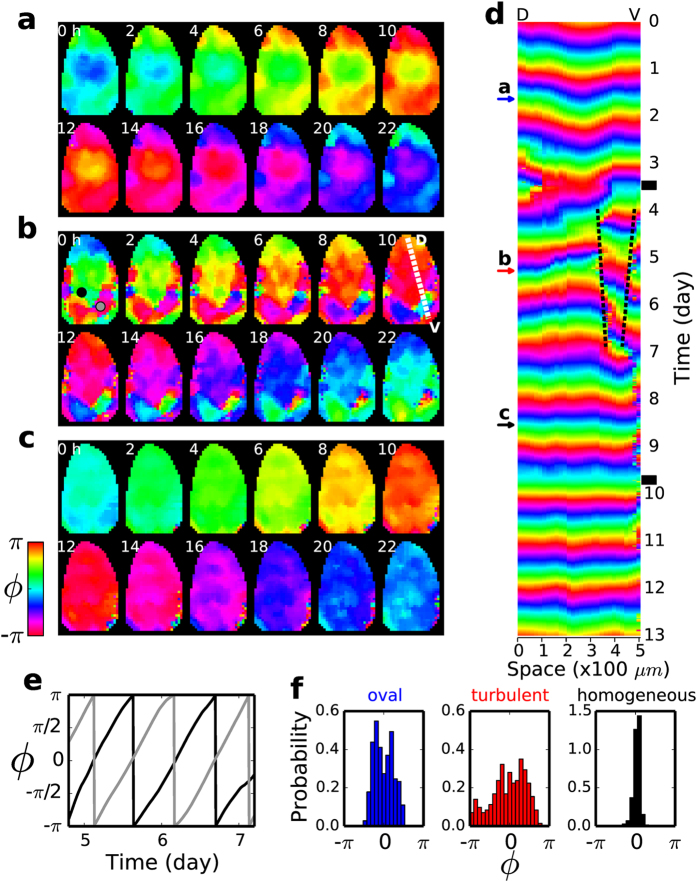
Multi-stability of the SCN phase wave rendered visible by temperature perturbations. (**a**) An oval shaped wave converging toward the middle of the nucleus. (**b**) A complex state having several fragmented domains oscillating out-of-phase to each other. (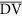 = 507*μm*) (**c**) An almost globally in-phase oscillation. (**d**) One dimensional space (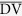) vs. time plot showing the formation of *phase bubbles*. (**e**) Two local time series oscillating anti-phase to each other: one obtained inside a phase bubble [location marked by a gray dot in (b, 0 h)] and the other just outside the bubble [black dot in (b, 0 h)]. (**f**) The histograms illustrating the difference in the degree of phase dispersal of the three different states, shown in (**a**), (**b**) and (**c**), respectively.

Shown in Fig. 3 is yet another type of SCN phase wave. A different SCN sample was monitored to support, more or less, an oval-shaped wave initially (Fig. 3a). Approximately, the wave started out from the perimeter of the SCN and traveled toward the central region, and the convergence is evident in the 1D space-time plot of Fig. 3d (day 0 ~ 3) as well as in Fig. 3a. We then applied a temperature perturbation, again when the range of phase dispersal was believed to include the unstable fixed point of the sample’s PRC. This time, instead of a pinwheel, the system produced several fragmented domains, some of which were oscillating almost completely out of phase with respect to its neighbor as shown in Fig. 3b,d,e (see Supplementary Movie 2) (similar dynamic domain structures were termed as “the phase bubbles” in^32^,^33^): Consequently, the phase dispersal in space almost became full coverage of the 2*π* range [see Fig. 3f (turbulent)] from the initial, narrower distribution [Fig. 3f (oval)]. Under the same (or similar) condition, phase bubbles were more popularly created (6 times out of 8 attempts, based on 6 different slices from six 6 different animals) than phase singularities (2 times out of 8 attempts). Unlike the case of the rotating pinwheel which also covers a full 2*π* range in space, however, the new complex state having phase bubbles was globally unstable and transformed to an almost phase-synchronized state as shown in Fig. 3c,f (homogeneous): in Fig. 3d, one shrinking phase bubble is delineated by a pair of black dashed lines. The transition had taken place by a rather abrupt disappearance of phase bubbles during day 7 ~ 8 (see Fig. 3d). The newly established phase-synchronized state was stable even after another perturbation was delivered (between day 9 and day 10).

### Desynchronization in the absence of action potentials

SCN clock cell populations are known to be quite heterogeneous as far as their endogenous periods are concerned^3^,^5^,^8^. Accordingly, several earlier studies emphasized the importance of cell-to-cell coupling agents as for maintaining a coherent circadian rhythm across the populations. Yet, their assessments were not about the spatio-temporal evolutions but more about the statistics regarding the amplitudes and periods of circadian oscillators^13^,^17^. Thus, we carefully analyzed how SCN wave dynamics would change if one of the key intercellular coupling mechanism was removed^15^. More explicitly, we tested the long-term effect of blocking the spiking activity of SCN clock cells by administering a low level of tetrodotoxin (0.5 *μ*M TTX), a sodium-channel blocker.

**Figure 4:**
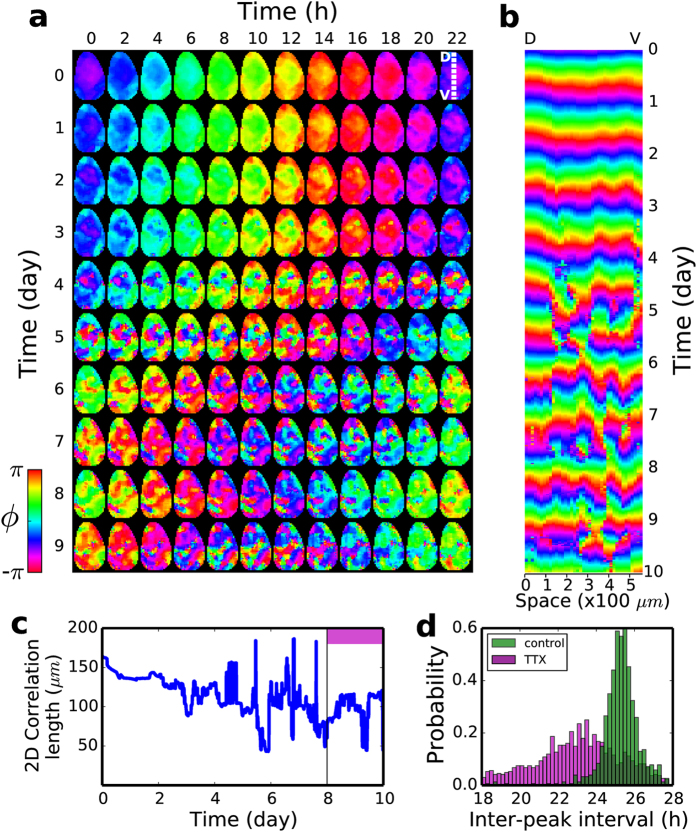
Transition to a phase turbulence in the presence of TTX. (**a**) A sequence of snapshots showing the gradual transition of an almost synchronized state to a phase turbulent state. (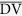 = 562*μm*) The culture medium in the control is switched to the one with TTX at (day 0, time 0). (**b**) A one-dimensional space (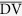) vs. time plot. (**c**) Moran’s 2-dimensional correlation length vs. the time with TTX. The Moran’s 2-dimensional spatial correlation function *I* was computed each time according to ref. 44 and fitted to a third order polynomial. Then, the first zero-crossing point of the fitted *I* was identified as the correlation length. (**d**) Inter-circadian peak-interval histogram, before (green) and after (violet) the TTX addition.

Shown in Fig. 4a is a sequence of snapshots showing temporal evolution toward a phase-desynchrony in detail (see Supplementary Movie 3). The initial state, which is nearly phase-synchronized, had been stable for more than a week when TTX was begun to be delivered (day 0, 0 h). With TTX present the circadian phase started to modulate in space. The degree of modulation increased gradually (see day 1 ~ 3) and eventually many small fragmented domains, oscillating out-of-phase, were brought about. The matching 1D space-time plot of Fig. 4b illustrates the desynchronizing process. Interestingly, the newly established dynamic state in the presence of TTX was not completely random but had a characteristic length (~100 μm), which was still much larger than the size of a single cell, as is shown in Fig. 4c (day 4 ~ 10). The erratic fluctuation in the correlation length during the TTX application could be attributed to the formation of new phase bubbles, their merging and decay. Also, rather significant is the broadening of the inter-peak interval distribution in the presence of TTX as shown in Fig. 4d. It originates not only from the decoupled oscillators recovering their own endogenous circadian rhythms but also from the complex spatio-temporal evolution of phase bubbles.

### Numerical simulations

**Figure 5:**
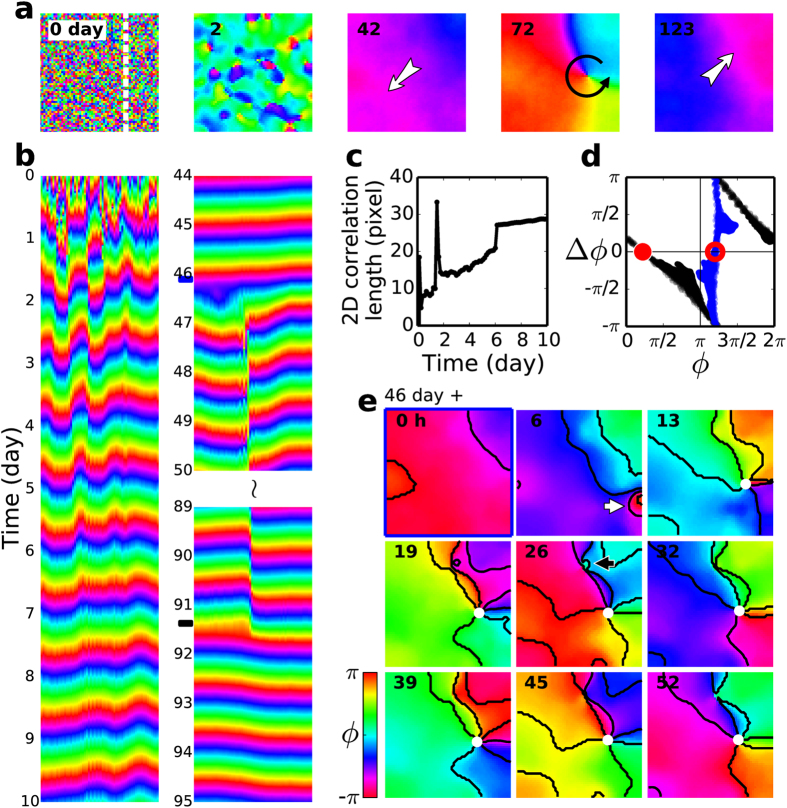
Formation of a pinwheel wave state and its removal by a global perturbation in a mathematical model of SCN. (**a**) Snapshot images of an uncoupled state (day 0), a transient state showing phase bubbles (day 2), a planar wave (moving toward the lower left-hand corner, day 42), a pinwheel wave (day 72), and another planar wave (moving toward the upper right-hand corner, day 123). (**b**) One-dimensional space-time plot illustrating the transition of an uncoupled random state to a coherent planar wave following an increase in the value of *K* to 1.8 from 0, the transition of a planar wave to a pinwheel state following a global perturbation (blue mark between day 46 and 47), and finally the transition back to a planar wave from the pinwheel state by another perturbation (black mark between day 91 and 92). (**c**) The increase of Moran’s 2-dimensional correlation length towards a coherent phase wave after the cell-to-cell coupling is turned on. (**d**) A PRC constructed based on the Δ*ϕ* measurements following two rounds of perturbations, one for the creation of the pinwheel (blue) and the other for the removal of it (black). (**e**) A sequence of snapshot images showing the formation of phase bubbles and phase singularities.

We could recapitulate most of our key experimental observations with numerical simulations and a mathematical model of an SCN that is described in *Methods*. Briefly, a two-dimensional 60 × 60 square grid was considered as an SCN, and each grid point was viewed as a grid point of the processed image of the experiment. The temporal evolution of each point was assumed to follow the seven-variable Becker-Weimann kinetics^34^ and neighboring cells were diffusively coupled to each other by a neurotransmitter which was considered to be an additional state variable. The endogenous periods of the model cells were made random (from 23.3 to 24.5 h)by choosing the value of *E* randomly from a uniform distribution. Initially, their phases were distributed randomly in space as shown in Fig. 5a (day 0). With no cell-to-cell coupling (*K* = 0), of course no spatial coherence existed. But, when *K* was changed to 1.8 at day 0, the coupled network started to organize, first into small synchronized domains (see Fig. 5a, day 2) oscillating out-of-phase relative to each other, and eventually into a single large planar wave (Fig. 5a, day 42; also, see Supplementary Movie 4). Obviously, the intercellular coupling brought a large-scale spatial coherence to the system in the form of a planar wave. Its gradual emergence is clear, not only in the one-dimensional space-time plot of Fig. 5b (day 0 ~ 10) but also in the correlation length shown in Fig. 5c. Interestingly, the overall behavior of the model system acquiring the phase coherence is opposite to the phase-destablizing process caused by TTX.

The temperature perturbation used in the experiment decreased the level of Per2 (see Supplementary Fig. S3). To introduce such an effect in the model simulation, we increased the value of parameter *k*_1*b*_ from 1.0 to 1.2 for 3 h, and that in turn decreased the level of Per2. As it was the case in the experiment, a properly timed homogeneous perturbation on a planar wave could lead to a rotating pinwheel state [see Fig. 5a (day 72) and Fig. 5b (day 46 ~ 91)] that covered a full 2*π* spectrum in 2D space for every instance of time. The time of the perturbation was chosen such that the range of *ϕ* covered by the initial planar wave would include the unstable fixed point of PRC, and Fig. 5d (blue dots) confirms that it was indeed the case with the unstable fixed point at ϕ^*^ = 1.2*π* (marked by an open red circle). As shown in Fig. 5e, with the dramatically increased phase dispersal following the perturbation and its own intrinsic inhomogeneities, the system produced two phase bubbles. One of them (marked by a white arrow) interacted with the existing planar wave to bring about a phase singularity (white dot), while the other smaller phase bubble (marked by a black arrow) got gradually entrained by the pinwheel wave (see Supplementary Movie 5). The perturbation-induced phase singularity was robust and persistent except for a transient initial drift (see Supplementary Fig. S4).

Another astonishing similarity with the experimental result was the removal of an existing phase singularity by the same perturbation used for its creation, as shown in Fig. 5a (day 123) and Fig. 5b (day 91 ~ 92; see Supplementary Movie 6). Again, it is worthwhile to note that just a single perturbation given to the pinwheel state is sufficient for the realization of the complete (flipped N-shaped) PRC as shown in Fig. 5d (black). Its similarity to those of experiments (Fig. 1d or Fig. 2c) is quite impressive. The resulting steady state was a planar wave [Fig. 5a (day 123), just like the one shown in Fig. 5a (day 42)], but moving in an almost opposite direction. In other words, the mathematical model system did not show any preference with regard to the direction of planar wave propagation.

## Discussion

In summary, we found that organotypically cultured *ex vivo* SCNs can support several different types of phase waves that are all long-term stable. Some of them were rendered possible by a finite temperature perturbation to which the system shows a strong nonlinear phase-shifting response. These results strongly suggest that neither specialized pacemaker nor topographically-fixed phase ordering exists within the SCN when it is cut out from the 24h afferent cues such as the daily light-dark cycle.

The phase singularity, about which a pinwheel wave was organized, could be created from a planar wave state by the temperature perturbation particularly when the wave’s phase dispersal in space covered the unstable domain (i. e., portion of PRC having a positive slope). Thereby, the existing phase dispersal greatly expanded to support a pinwheel covering the full 2*π* range. Interestingly, the period of the pinwheel wave (26.5 h) was notably longer than that of the planar wave (23.5 h) from which it was originated. The emergence of phase singularity, however, was not always guaranteed for a given similar situation; it seemed to depend on the degree of intrinsic inhomogeneity as well as the state (shape and degree of phase dispersal) of the existing wave. Often, phase bubbles could be born out of the same perturbation instead. Once created, the pinwheel state was very robust while the bubble state was unstable. Interestingly, we could also remove the pinwheel using the same perturbation. Indeed, due to the non-monotonic nature of clock cell PRC, the homogeneous perturbation could not only shift but also expand or shrink the existing phase dispersal to result in a different wave morphology.

Using a mathematical model, we successfully reproduced most of our experimental findings, including the flipped N-shaped PRC, rotating pinwheel and other types of waves, and the effect of a cell-to-cell decoupling. The analogy between the experiments and numerical simulations is rather compelling, especially, the phenomenon that a properly-timed perturbation leads to a rotating pinwheel from an initial planar wave state, and another perturbation brings a planar wave back from the pinwheel state.

The equi-phase contours associated with the planar and pinwheel wavefronts are neither smooth nor stationary as shown in Fig. 2, and it may reflect the peptidergic separation within the SCN like the core region, characterized by gastrin-releasing peptide and vasoactive intestinal peptide, and the shell region, bearing arginine-vasopressin^9^,^10^. Besides, we find that the SCN retains a sizable (~100 μm) correlation length even in the presence of TTX, and this may be a clue for the existence of yet-to-be-identified smaller sub-structures within the SCN. Alternatively, it could be an indication that there still exists some short-range cell-to-cell interaction mediated by diffusing peptides. Moreover, some localized spatial inhomogeneities could have played a role in producing both phase singularities and bubbles, since for numerical simulations the pinwheel state could not be realized without some spatial inhomogeneities. On the other hand, our study suggests that there exists no specialized organizing center (i. e. pacemaker) within an SCN orchestrating the phase dynamics, because different phase waves were found to travel stably in various directions with different shapes.

From a mathematical point of view the systems under study are multi-stable (i.e., more than 2 stable states can exist for a given condition). For an example, see the simulation result shown in Fig. 5a: planar wave moving to the upper-right corner (t = 42), pinwheel (t = 72), planar wave moving toward the lower-left corner (t = 123) and homogeneous oscillation (not shown). Thus, with several different states being available, in principle many different transitions are possible, and the outcome of a perturbation given to a particular state will depend on the nature of the perturbation (eg. amplitude, duration, timing, etc) and the specific dynamic phase of the system when it was perturbed. In other words, without a comprehensive knowledge about the system, a definite prediction on the type of transition will not be possible. In a way, our current paper focuses at two particular transitions: namely, the one from a planar wave to a pinwheel wave and its reverse (Fig. 2 and Fig. 5). Under a similar condition “phase bubbles” emerge more frequently than a pinwheel, but they were only transient, as discussed with Fig. 3. Phase bubbles were seemed stable only for the TTX-treated case of Fig. 4.

Essentially, the PRC is a property of a single (cell) oscillator and its shape reflects the form of the underlying limit-cycle attractor^35^ and the flow around it in phase space. In chronobiology, however, it has been a normal practice to discuss the PRC of a *system* like the whole SCN or even a group of animals, assuming there is only one phase representing the whole population^1^,^35^,^36^. There will be no trouble with this practice, as long as the constituents which comprise the system share and maintain the same or a similar phase and lie in the stable regime of PRC away from its repeller fixed point. But, this study clearly shows that there can be non-trivial spatio-temporal phase dynamics within an SCN so that defining a single (eg. arithmetic mean) phase or phase shift can be ambiguous, if not meaningless. For example, we cannot assign a single representative phase either for the pinwheel state or for the complex state filled with phase bubbles. In addition, SCN clock cells can be non-uniform as far as their PRCs are concerned. We note that the PRC data points in Fig. 2c (or Fig. 1d), which are constructed based on many different cells comprising the system, scatter significantly. The difference between the PRCs of different SCNs (animals) can be even more significant. For example, if we compare the position of stable (or unstable) fixed point of the PRC of Fig. 1d and that of Fig. 2c, the difference is nearly *π*/2. The same is true with numerical simulations: The shape of PRC can be easily altered by changing some parameter values affecting the limit-cycle attractor and the flow around it; moreover, even for a given system, the degree of phase dispersal and morphology of the phase wave can influence the shape of the PRC significantly.

There are several inconsistent reports on the effects of a similar temperature perturbation given to SCN slices. Briefly, some years ago Ruby *et al.*^26^ reported that the circadian phases of 3 to 4 months old rats could be changed by a 2-hr, 34 → 37 °C temperature pulse. Herzog *et al.*^27^ also reported that the circadian rhythms of adult as well as young rats are responsive to a temperature modulation. However, Buhr *et al.*^28^ claimed that the PER2 circadian bioluminescence rhythms of adult mouse SCN explants did not respond to a similar temperature perturbation (6-hr, 36.0 → 38.5 °C pulse) unless the action potential mediated cell-to-cell connectivity was compromised. Thus, the temperature perturbation effect became an issue of controversy. Buhr *et al.* suggested that the discrepancy could be due to the age difference in the animals being used^28^. But Ruby claimed that it could not be the case^37^. Buhr suggested an alternative possibility that the discrepancy originates from the difference between rats and mice^38^. More recently, however, an independent study by Bechtold *et al.* reported an extensive set of data revealing a non-monotonic PRC of fully grown (10–16 week old) mice SCN explants^36^. The PRC was based on a very similar temperature perturbation (2-hr, 36.0 → 38.5 °C pulse) and it is quite similar to what we report in the current work. After all, the age seems not be a key determining factor as far as the temperature perturbation effects are concerned. In any case, the aim of this paper is not to resolve the reported discrepancy. In our case, the 6-hr, 36.0 → 38.5 °C pulse was effective and strong enough for revealing the hidden multi-stability.

The SCN has at least several different efferent connections to various areas in the brain^39^,^40^ to control different biological rhythms which normally run with a well-maintained relative phase difference to the other: for example, the body temperature rises in the early morning, whereas the level of sleep hormone melatonin goes high in the late evening. Although the information regarding where and how exactly these efferent connections project out from the SCN is still lacking, it is a general presumption that they are spatially distributed throughout the nucleus. If this is the case, the targets of efferent projections can receive a rather different relative phase information once the mode of phase wave within the SCN changes from one type to another. Some unusual modes of waves can underlie serious circadian rhythm disorders. Incidentally, the visual similarity between the SCN circadian pinwheel and the cardiac pinwheel wave (termed “the reentry”) underlying the cardiac arrhythmia called tachycardia^41^ is rather striking, and this may suggest that the circadian pinwheel is perhaps a pathological dynamic state of an SCN.

Several interesting questions have emerged out of this study. What is the degree of phase dispersal (or wavelength) that is normally or can be supported by SCN and what is the relevant wave dispersion relation (i. e., phase velocity vs. wavelength) like? Also, there can be non-trivial spatio-temporal dynamics and instabilities associated with the pinwheel state^31^. Obviously, characterizing the complex phase dynamics specific to SCN will be essential for understanding the inner-workings of the clock and various biomedical issues associated with it.

## Methods

### SCN slice culture

Transgenic knock-in PER2::LUC mice (2 ~ 3 day old) were frozen to death within 5 minutes. Their brains were then quickly removed and immersed in a dish containing ice-cold slush of oxygenated 95% O_2_, 5% CO_2_ dissecting solution (in mM: 138.6 NaCl, 3.35 KCl, 0.6 NaH_2_PO_4_, 21.0 NaHCO_3_, 9.9 D-glucose, 0.5 CaCl_2_, 5.0 MgSO_4_). Coronal hypothalamic slices (300) *μ*m in thickness) containing a pair of SCN nuclei were cut as described in ref. 13. Then, the slices containing the central part of the SCN were selected, picked up using a pipette (~8 mm stem dia.), transferred onto the membrane of a 0.4 *μ*m culture plate insert (PICM03050, Millicell-CM, Millipore, MA, USA), placed in a standard 6-well plate (140675, Nunc) and cultured with 1 mL of medium that consisted of 57% heat-inactivated horse serum (Gibco, USA), 25 g/mL d-glucose (Sigma-Aldrich, USA), 14% Eagle’s balanced salt solution (Gibco, USA), 1x glutamax (glutamax-I, Gibco, USA), kanamycin (Gibco, USA), 28% Eagle’s basal medium (Gibco, USA). About two weeks later, a part of the membrane around a selected SCN slice was severed and transferred onto a collagen-coated dish upside down. A very small amount (180 *μ*l) of the culture medium was added just enough to soak the SCN tissue. The dish was covered with a thin (0.2 mm thickness) polydimethylsiloxane (PDMS, Sylgard 184, Dow Corning, USA) membrane, which has very good CO_2_ and O_2_ permeability, to prevent evaporation.

This study was approved by our institutional review board for animal research, the Korea University Institutional Animal Care and Use Committee (KUIACUC-2011-197). The methods were carried out in accordance with the approved guidelines.

### Tetrodotoxin application

We used TTX to block the intercellular communication by synaptic coupling and to see how that affected the phase relationships among SCN cells. Stock solution (1 mM TTX, Sigma, USA) was made with phosphate-buffered saline and a working solution (0.5 *μ*M) was prepared by diluting the stock solution with SCN culture medium.

### Experimental setup

For a long-term PER2::LUC bioluminescence imaging, a thin cylindrical chamber was designed and built as shown in Fig. 1a. An insulated heating wire heated the water inside the chamber, and the temperature inside was monitored by a thermometer (PT100, San-up electronics, South Korea), actively regulated at 36.0 ± 0.1 °C by a temperature controller (SDM9000, Sanup, Korea), and logged every second. The relaxation time constants for the increase (36.0 → 38.5 °C) [and the decrease (38.5 → 36.0 °C)] are measured to be 0.8 hr and 1.5 hr, respectively. Two ITO-coated optical windows along the imaging axis were also electrically heated to prevent water condensation that would otherwise obstruct the imaging. A gas mixture (5% CO_2_, 95% air) was continuously bubbled in through the water bath.

When an SCN sample was ready for experiment, the culture medium was replaced by the one containing 0.1 mM firefly luciferin (P1041, Promega, USA). Then, the sample was loaded into the chamber, and the chamber was positioned on a microscope stage (IX71, Olympus, Japan). The PER2::LUC bioluminescence was collected by a large numerical aperture (NA 0.40), long working distance (3.1 mm) objective lens (UPLSAPO 10X, Olympus, Japan) and images were captured via an EMCCD camera (iXon DU860, Andor, Northern Ireland, UK) mounted on the side port of the microscope through a C-mount coupler (0.25x, Olympus, Japan). Each (128 × 128 pixels) image was integrated for 9 minutes and one was taken every 10 minutes with a EM gain of 80% and a read-out rate of 1 MHz via Andor Solis imaging software. The culture medium was changed every 2 ~ 3 days manually on a clean bench within 5 minutes, so as to minimize the unavoidable temperature drop. This short-term, interruption was not at all significant compared to the 6-h long temperature perturbation.

### Image analysis

The images were acquired once every 10 minutes. The entire image sequence for a successful experimental run often included more than 10 image stacks, each of which covered a duration of 2 ~ 3 days between two consecutive culture medium changes. Each medium change necessitated an inevitable sample repositioning. Subsequently, different image stacks were aligned and concatenated together using a customized “StackReg” ImageJ plugin (Biomedical Imaging Group, NIH).

Since the luminescence signal was very weak and noisy, a spatial 3 × 3 binning was applied during image processing for a better signal-to-noise ratio. The noisy amplitude time trace of each pixel in the reduced image was filtered first by fitting neighboring data points of 12 h locally to a third-order polynomial and then detrended by subtracting the moving average of 24 h window of the smoothed data. Finally, the filtered time series *x*(*t*) was used for a Hilbert transformation, 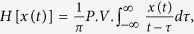 which was then used to define an analytic signal *x*_*a*_(*t*) = *x*(*t*) + *iH*(*t*) = *A*(*t*)*e*^*iϕ*(*t*)^^25^. The amplitude peak positions, corresponding to *ϕ* = 0, were determined by fitting 6 points around each phase zero-crossing to a straight line and using interpolation.

### Mathematical model SCN

Our mathematical model of an SCN is a two-dimensional lattice of 60 × 60 grid points and each point can be considered as a small cluster of clock cells oscillating in synchrony. Each grid point oscillates through interlocked transcriptional and translational feedback loops of seven variables, *Y*_1_, *Y*_2_, …, *Y*_7_, which represent the concentrations of Per2 (or Cry) mRNA, PER2/CRY complex in cytoplasm, PER2/CRY complex in nucleus, Bmal1 mRNA, BAML1 protein in cytoplasm, BAML1 protein in nucleus, BMAL1/CLOCK complex or phosphorylated form of BMAL1, respectively^34^. Then, we have assumed a diffusive cell-to-cell coupling: Each cell is coupled to its four nearest neighbors such that the amount of neurotransmitter (*Y*_8_) produced by them influences the Per2 mRNA (*Y*_1_) transcription rate of the corresponding oscillator. Then, a four-neighbor, weighted-sum of *Y*_8_ is added to the equation of *Y*_1_ as given in the paper^42^. Our model equations are:


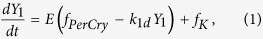



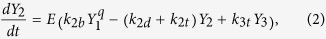



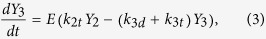



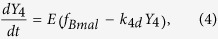



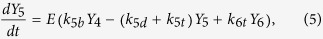



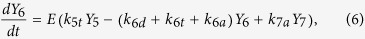



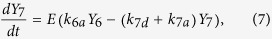



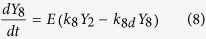


where


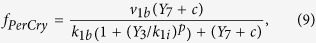



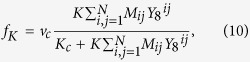



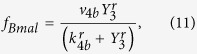


and the coupling matrix


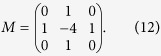


The following set of fixed parameter values were used throughout this paper: *v*_1*b*_ = 9.0 nMh^−1^; *k*_1*b*_ = 1.0 nM; *k*_1*i*_ = 0.56 nM; *c* = 0.01 nM; *p* = 5.0; *k*_1*d*_ = 0.12 h^−1^; *k*_2*b*_ = 0.3 nM^−1^h^−1^; *q* = 2.0; *k*_2*d*_ = 0.05 h^−1^; *k*_2*t*_ = 0.24 h^−1^; *k*_3*t*_ = 0.02 h^−1^; *k*_3*d*_ = 0.12 h^−1^; *v*_4*b*_ = 3.6 nMh^−1^; *k*_4*b*_ = 2.16 nM; *r* = 3.0; *k*_4*d*_ = 0.75 h^−1^; *k*_5*b*_ = 0.24 h^−1^; *k*_5*d*_ = 0.06 h^−1^; *k*_5*t*_ = 0.45 h^−1^; *k*_6*t*_ = 0.06 h^−1^; *k*_6*d*_ = 0.12 h^−1^; *k*_6*a*_ = 0.09 h^−1^; *k*_7*a*_ = 0.003 h^−1^; *k*_7*d*_ = 0.09 h^−1^; *k*_8_ = 1.0 h^−1^; *k*_8*d*_ = 4.0 h^−1^; *v*_*c*_ = 0.68 nMh^−1^; and *K*_*c*_ = 4.8 h^−1^. To emulate some cell-to-cell circadian period variation (23.3 ~ 24.5 h), for each grid point we used a different *E* value that was randomly chosen from a uniform distribution (0.259 ~ 0.273). Also, the initial values of *Y*_*i*_ were chosen randomly from a uniform distribution (1.0 ~ 3.0). The set of equations was integrated using FORTRAN odepack with no flux boundary condition.

## Additional Information

**How to cite this article**: Jeong, B. *et al.* Multi-stability of circadian phase wave within early postnatal suprachiasmatic nucleus. *Sci. Rep.* **6**, 21463; doi: 10.1038/srep21463 (2016).

## Supplementary Material

Supplementary Information

Supplementary Movie S1

Supplementary Movie S2

Supplementary Movie S3

Supplementary Movie S4

Supplementary Movie S5

Supplementary Movie S6

## References

[b1] Klein D. C., Moore R. Y. & Reppert S. M. Suprachiasmatic Nucleus: The Mind’s Clock (Oxford, 1991).

[b2] Reppert S. M. & Weaver D. R. Coordination of circadian timing in mammals. Nature 418, 935–941 (2002). 12198538 10.1038/nature00965

[b3] Welsh D. K., Logothetis D. E., Meister M. & Reppert S. M. Individual neurons dissociated from rat suprachiasmatic nucleus express independently phased circadian firing rhythms. Neuron 14, 697–706 (1995). 7718233 10.1016/0896-6273(95)90214-7

[b4] Ikeda M. *et al.* Circadian dynamics of cytosolic and nuclear Ca2+ in single suprachiasmatic nucleus neurons. Neuron 38, 253–263 (2003). 12718859 10.1016/s0896-6273(03)00164-8

[b5] Honma S., Nakamura W., Shirakawa T. & Honma K. Diversity in the circadian periods of single neurons of the rat suprachiasmatic nucleus depends on nuclear structure and intrinsic period. Neurosci. Lett. 358, 173–176 (2004). 15039109 10.1016/j.neulet.2004.01.022

[b6] Antle M. C. & Silver R. Orchestrating time: arrangements of the brain circadian clock. Trends Neurosci. 28, 145–151 (2005). 15749168 10.1016/j.tins.2005.01.003

[b7] Colwell C. S. Linking neural activity and molecular oscillations in the SCN. Nat. Rev. Neurosci. 12, 553–569 (2011). 21886186 10.1038/nrn3086 PMC4356239

[b8] Welsh D. K., Takahashi J. S. & Kay S. A. Suprachiasmatic nucleus: cell autonomy and network properties. Annu. Rev. Physiol. 72, 551–577 (2010). 20148688 10.1146/annurev-physiol-021909-135919 PMC3758475

[b9] Lokshin M., LeSauter J. & Silver R. Selective distribution of retinal input to mouse SCN revealed in analysis of sagittal sections. J. Biol. Rhythms. 30, 251–257 (2015). 25994103 10.1177/0748730415584058 PMC4933594

[b10] Bedont J. L. & Blackshaw S. Constructing the suprachiasmatic nucleus: a watchmaker’s perspective on the central clockworks. Front. Syst. Neurosci. 9, 74 (2015). 26005407 10.3389/fnsys.2015.00074 PMC4424844

[b11] Aton S. J., Colwell C. S., Harmar A. J., Waschek J. & Herzog E. D. Vasoactive intestinal polypeptide mediates circadian rhythmicity and synchrony in mammalian clock neurons. Nat. Neurosci. 8, 476–483 (2005). 15750589 10.1038/nn1419 PMC1628303

[b12] Liu A. C. *et al.* Intercellular coupling confers robustness against mutations in the SCN circadian clock network. Cell 129, 605–616 (2007). 17482552 10.1016/j.cell.2007.02.047 PMC3749832

[b13] Hong J. H., Jeong B., Min C. H. & Lee K. J. Circadian waves of cytosolic calcium concentration and long-range network connections in rat suprachiasmatic nucleus. Eur. J. Neurosci. 35, 1417–1425 (2012). 22501027 10.1111/j.1460-9568.2012.08069.x

[b14] Freeman G. M., Krock R. M., Aton S. J., Thaben P. & Herzog E. D. GABA networks destabilize genetic oscillations in the circadian pacemaker. Neuron 78, 799–806 (2013). 23764285 10.1016/j.neuron.2013.04.003 PMC3683151

[b15] Jones J. R., Tackenberg M. C. & McMahon D. G. Manipulating circadian clock neuron firing rate resets molecular circadian rhythms and behavior. Nat. Neuros. 18, 373–375 (2015). 10.1038/nn.3937 PMC4502919 25643294

[b16] Pikovsky A., Rosenblum M. & Kurths J. Synchronization a Universal Concept in Nonlinear Sciences, (Cambridge UP, Cambridge, 2003).

[b17] Yamaguchi S. *et al.* Synchronization of cellular clocks in the suprachiasmatic nucleus. Science 302, 1408–1412 (2003). 14631044 10.1126/science.1089287

[b18] Quintero J. E., Kuhlman S. J. & Mcmahon D. G. The biological clock nucleus: a multiphasic oscillator network regulated by light. J. Neurosci. 23, 8070–8076 (2003). 12954869 10.1523/JNEUROSCI.23-22-08070.2003 PMC6740506

[b19] Foley N. C. *et al.* Characterization of orderly spatiotemporal patterns of clock gene activation in mammalian suprachiasmatic nucleus. Eur. J. Neurosci. 33, 1851–1865 (2011). 21488990 10.1111/j.1460-9568.2011.07682.x PMC3423955

[b20] Evans J. A., Leise T. L., Castanon-Cervantes O. & Davidson A. J. Intrinsic regulation of spatiotemporal organization within the suprachiasmatic nucleus. PLoS ONE 6, e15869 (2011). 21249213 10.1371/journal.pone.0015869 PMC3017566

[b21] Enoki R., Ono D., Hasan M. T., Honma S. & Honma K. Topological specificity and hierarchical network of the circadian calcium rhythm in the suprachiasmatic nucleus. Proc. Natl. Acad. Sci. USA 109, 21498–21503 (2012). 23213253 10.1073/pnas.1214415110 PMC3535646

[b22] Brancaccio M., Maywood E. S., Chesham J. E., Loudon A. S. & Hastings M. H. A Gq-Ca2+ axis controls circuit-level encoding of circadian time in the suprachiasmatic nucleus. Neuron 78, 714–728 (2013). 23623697 10.1016/j.neuron.2013.03.011 PMC3666084

[b23] Mieda M. *et al.* Cellular clocks in AVP neurons of the SCN are critical for interneuronal coupling regulating circadian behavior rhythm. Neuron 85, 1103–1116 (2015). 25741730 10.1016/j.neuron.2015.02.005

[b24] Lee I. T. *et al.* Neuromedin s-producing neurons act as essential pacemakers in the suprachiasmatic nucleus to couple clock neurons and dictate circadian rhythms. Neuron 85, 1086–1102 (2015). 25741729 10.1016/j.neuron.2015.02.006 PMC5811223

[b25] Le Van Quyen M. *et al.* Comparison of Hilbert transform and wavelet methods for the analysis of neuronal synchrony. J. Neurosci. Meth. 111, 83–98 (2001). 10.1016/s0165-0270(01)00372-7 11595276

[b26] Ruby N. F., Burns E. D. & Heller H. C. Circadian rhythms in the suprachiasmatic nucleus are temperature-compensated and phase-shifted by heat pulses *in vitro*. J. Neurosci. 19, 8630–8636 (1999). 10493763 10.1523/JNEUROSCI.19-19-08630.1999 PMC6783024

[b27] Herzog E. D. & Huckfeldt R. M. Circadian entrainment to temperature, but not light, in the isolated suprachiasmatic nucleus. J. Neurophysiol. 90, 763–770 (2003). 12660349 10.1152/jn.00129.2003

[b28] Buhr E. D., Yoo S. H. & Takahashi J. S. Temperature as a universal resetting cue for mammalian circadian oscillators. Science 330, 379–385 (2010). 20947768 10.1126/science.1195262 PMC3625727

[b29] Sinha S. & Sridhar S. Patterns in Excitable Media, (CRC Press, New York, 2015).

[b30] Doi M. *et al.* Circadian regulation of intracellular G-protein signalling mediates intercellular synchrony and rhythmicity in the suprachiasmatic nucleus. Nat. Commun. 2, 327 (2011). 21610730 10.1038/ncomms1316 PMC3112533

[b31] Hagberg E. & Meron E. From labyrinthine patterns to spiral turbulence. Phys. Rev. Lett. 72, 2494–2497 (1994). 10055894 10.1103/PhysRevLett.72.2494

[b32] Moon S. J. *et al.* Phase bubbles and spatiotemporal chaos in granular patterns. Phys. Rev. E 65, 011301 (2001). 10.1103/PhysRevE.65.011301 11800687

[b33] Park J.-S., Woo S.-J., Kwon O., Kim T. Y. & Lee K. J. Nucleation, drift, and decay of phase bubbles in period-2 oscillatory wave trains in a reaction-diffusion system. Phys. Rev. Lett. 100, 068302 (2008). 18352523 10.1103/PhysRevLett.100.068302

[b34] Becker-Weimann S., Wolf J., Herzel H. & Kramer A. Modeling feedback loops of the mammalian circadian oscillator. Biophys. J. 63, 3023–3034 (2004). 15347590 10.1529/biophysj.104.040824 PMC1304775

[b35] Johnson C. H. Forty years of PRCs–what have we learned? Chronobiol. Intl. 15, 711–743 (1999). 10.3109/07420529909016940 10584173

[b36] Pilorz V. *et al.* A novel mechanism controlling resetting speed of the circadian clock to environmental stimuli. Curr. Biol. 24, 766–773 (2014). 24656826 10.1016/j.cub.2014.02.027 PMC3988889

[b37] Ruby N. F. Rethinking temperature sensitivity of the suprachiasmatic nucleus. J. Biol. Rhythms 26, 368–370 (2011). 21775296 10.1177/0748730411411678

[b38] Buhr E. D., Yoo S. H. & Takahashi J. S. Phase-resetting sensitivity of the suprachiasmatic nucleus and oscillator amplitude reply to letter by ruby. J. Biol. Rhythms 26, 371–373 (2011).

[b39] Abrahamson E. E. & Moore R. Y., Suprachiasmatic nucleus in the mouse: retinal innervation, intrinsic organization and efferent projections. Brain Res. 916, 172–191 (2001). 11597605 10.1016/s0006-8993(01)02890-6

[b40] Kriegsfeld L. J., Leak R. K., Yackulic C. B., LeSauter J. & Silver R. Organization of suprachiasmatic nucleus projections in syrian hamsters (mesocricetus auratus): an anterograde and retrograde Analysis. J. Comp. Neurol. 468, 361–379 (2004). 14681931 10.1002/cne.10995 PMC3275427

[b41] Davidenko J. M., Pertsov A. V., Salomonsz R., Baxter W. & Jalife J. Stationary and drifting spiral waves of excitation in isolated cardiac muscle. Nature 355, 349–351 (1992). 1731248 10.1038/355349a0

[b42] Bernard S., Gonze D., Čajavec B., Herzel H. & Kramer A. Synchronization-induced rhythmicity of circadian oscillators in the suprachiasmatic nucleus. PLoS Comput. Biol. 3, e68 (2007). 17432930 10.1371/journal.pcbi.0030068 PMC1851983

[b43] Iyer A. N. & Gray R. A. An experimentalist’s approach to accurate localization of phase singularities during reentry. Ann. Biomed. Eng. 29, 47–59 (2001). 11219507 10.1114/1.1335538

[b44] Legendre P. & Legendre L. Numerical ecology, second English edition, (Elsevier, Amsterdam, 1998).

