# Corrigendum: Multi-stability of circadian phase wave within early postnatal suprachiasmatic nucleus

**DOI:** 10.1038/srep25430

**Published:** 2016-05-09

**Authors:** Byeongha Jeong, Jin Hee Hong, Hyun Kim, Han Kyoung Choe, Kyungjin Kim, Kyoung J. Lee

Scientific Reports 6: Article number: 21463;10.1038/srep21463 Published online 02192016; Updated 05092016

The original version of this Article contained errors in the Abstract.

“In different SCN cells, oscillations of biochemical markers such as the expression-level of clock genes, are not synchronized but instead form slow circadian phase waves propagating over the whole cell population spatio-temporal struc- ture is a fixed property set by the anatomy of a given SCN. Here, we show that this is not the case in early postnatal SCN. Earlier studies presumed that their”.

now reads:

“In different SCN cells, oscillations of biochemical markers such as the expression-level of clock genes, are not synchronized but instead form slow circadian phase waves propagating over the whole cell population. Earlier studies presumed that their spatio-temporal structure is a fixed property set by the anatomy of a given SCN. Here, we show that this is not the case in early postnatal SCN”.

These errors have now been corrected in the PDF and HTML versions of the Article.

