# Supplementary material for: Multi-stability of circadian phase wave within early postnatal suprachiasmatic nucleus
Source: Sci Rep. 2016 Feb 19;6:21463. doi: 10.1038/srep21463 (PMC4759822; doi:10.1038/srep21463)
Supplement: Supplementary Information [file srep21463-s1.pdf]

Multi-stability of circadian phase wave within early postnatal suprachiasmatic nucleus

Byeongha Jeong[1], Jin Hee Hong[1], Hyun Kim[1], Han Kyoung Choe[2], Kyungjin Kim[2], Kyoung J. Lee[1*]

[1]*Department of Physics, Korea University, Seoul 136-713, Korea*

[2]*School of Biological Sciences, Seoul National University, Seoul 151-742, Korea*

*Corresponding author: kyoung@korea.ac.kr

SUPPLEMENTARY FIGURES

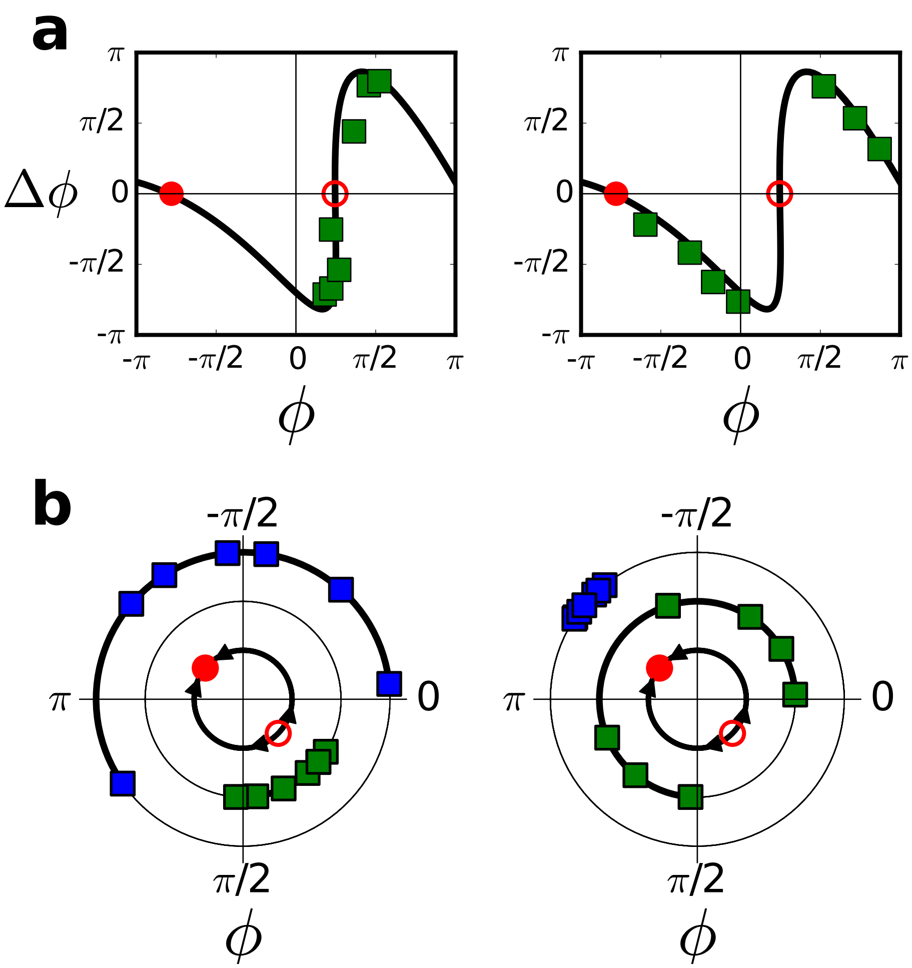

Supplementary Figure 1. **Schematic illustration of the effect of a perturbation on the phase dispersal**: (a) Broadening when the initial phases (green squares) all lie in the unstable branch, where the PRC slope is positive, and (b) Tightening when the initial phases (green squares) all lie in the stable branch, where the PRC slope is negative. Blue squares represent the phases after each perturbation. Red filled (open) circle represents the stable (unstable) fixed point. The bottom row illustrates two different effects of a perturbation (inner circle: before, outer circle: after) matching the two different modes of initial phase dispersal on the top row.

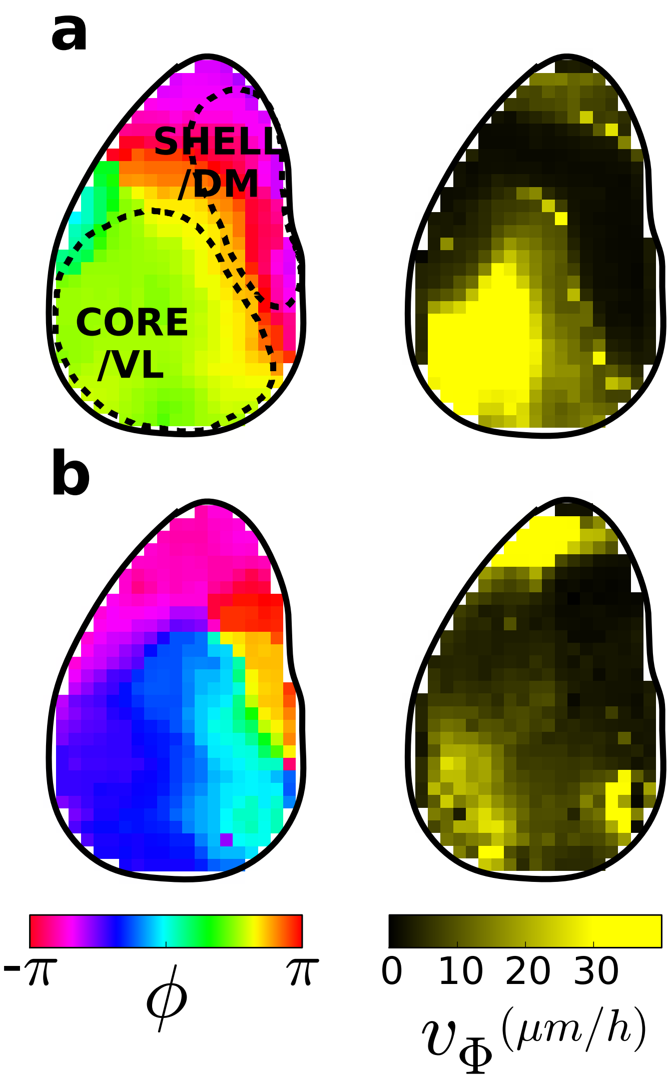

Supplementary Figure 2. **Local phase velocity maps for the crescent-shaped wave (a) and pinwheel wave (b).** The instantaneous phase velocity maps (right column) were calculated according to $v_\Phi = (\frac{\partial \Phi}{\partial t})|\nabla \phi|^{-1}$. Shown in the left column are two exemplary phase maps.

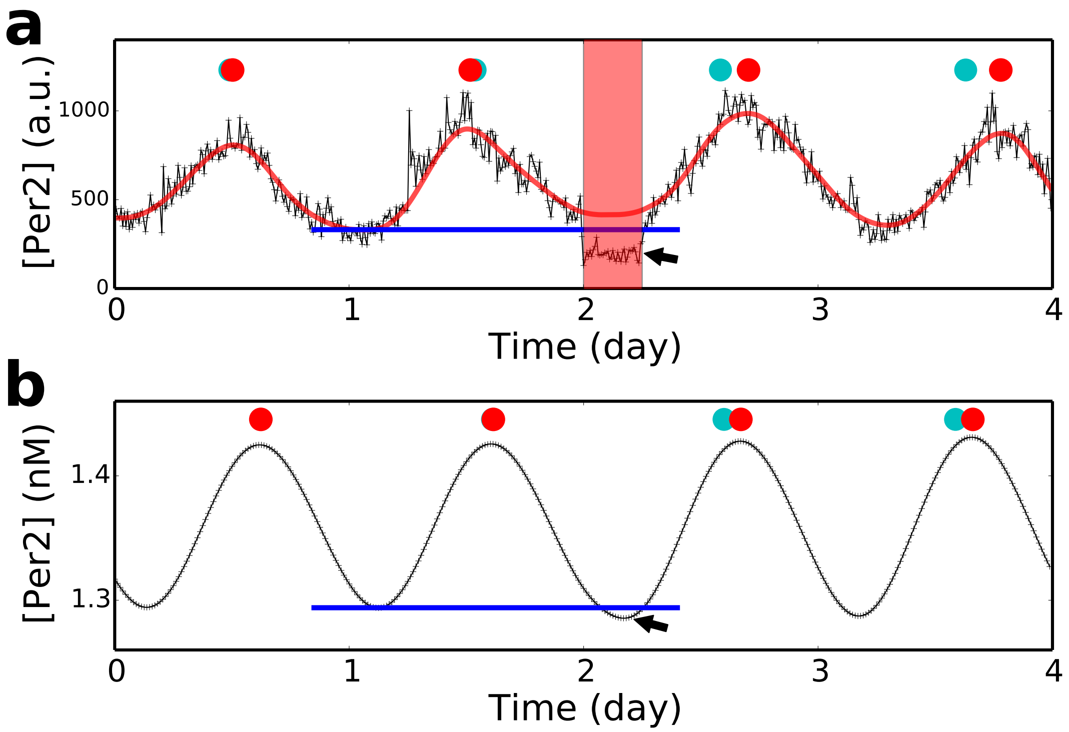

Supplementary Figure 3. **Decreased level of [Per2] during a perturbation in experiment (a) and model simulation (b).**

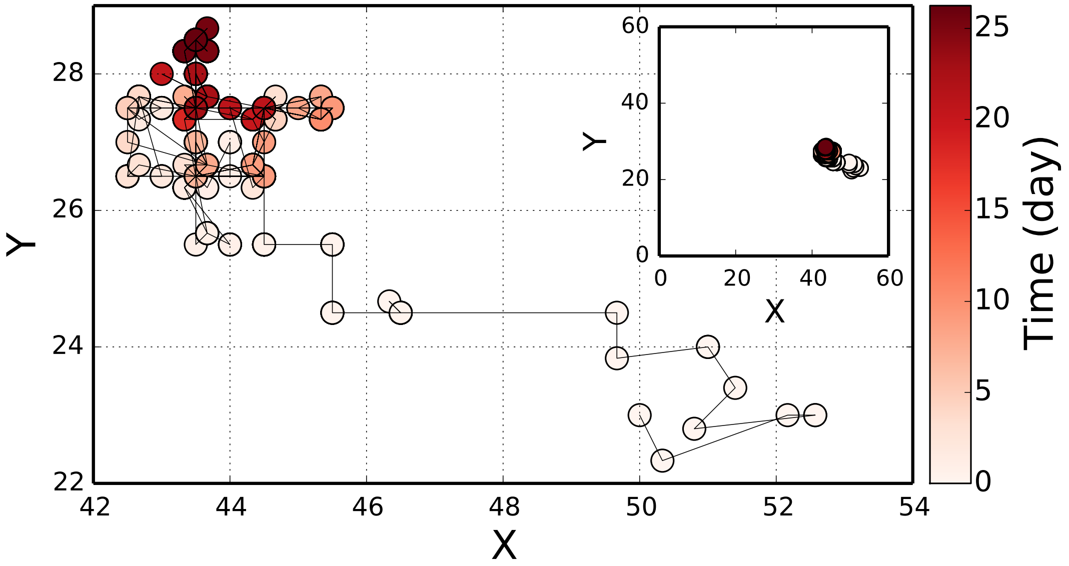

Supplementary Figure 4. **The drift of a phase singularity during an initial transient period after its creation.**

SUPPLEMENTARY MOVIE LEGENDS

- Supplementary Movie 1: The perturbation-induced birth and death of a circadian pinwheel wave. The circadian variation of [Per2] level in an SCN is presented: initially in a form of crescent wave, then a pinwheel wave after a temperature perturbation (TS1), and finally a new state of almost perfect synchrony, subsequent to another temperature perturbation (TS2). See Fig. 2a for its matching sequence of snapshots.
- Supplementary Movie 2: The creation of phase bubbles and their subsequent decay to a synchronous state. The video shows an oval shaped wave transforming, first to a state with phase bubbles, then to a synchronous state, both subsequent to a temperature perturbation (TS1). Also shown is a stable synchronous state, not being affected by an additional perturbation (TS2).
- Supplementary Movie 3: The destablization of a coherent circadian phase wave into a turbulent state following a TTX application.
- Supplementary Movie 4: The emergence of a coherent circadian planar wave from an initial phase-incoherent state (numerical simulation of an SCN model).
- Supplementary Movie 5: The emergence of a pinwheel wave state following a global perturbation given to a planar wave (numerical simulation of an SCN model).
- Supplementary Movie 6: The termination of a pinwheel wave state following a global perturbation, leading to another planar wave (numerical simulation of an SCN model).
